# Collagen Family Genes Associated with Risk of Recurrence after Radiation Therapy for Vestibular Schwannoma and Pan-Cancer Analysis

**DOI:** 10.1155/2021/7897994

**Published:** 2021-10-13

**Authors:** Qingyuan Shi, Xiaojun Yan, Jiyun Wang, Xiangbao Zhang

**Affiliations:** ^1^Department of Otolaryngology, HwaMei Hospital, University of Chinese Academy of Sciences, China; ^2^Department of Otorhinolaryngology Head and Neck Surgery, Shanghai Ninth People's Hospital, Shanghai Jiao Tong University School of Medicine, Shanghai, China

## Abstract

**Background:**

The safety of radiotherapy techniques in the treatment of vestibular schwannoma (VS) shows a high rate of tumor control with few side effects. Neuropeptide Y (NPY) may have a potential relevance to the recurrence of VS. Further research is still needed on the key genes that determine the sensitivity of VS to radiation therapy.

**Materials and Methods:**

Transcriptional microarray data and clinical information data from VS patients were downloaded from GSE141801, and vascular-related genes associated with recurrence after radiation therapy for VS were obtained by combining information from MSigDB. Logistics regression was applied to construct a column line graph prediction model for recurrence status after radiation therapy. Pan-cancer analysis was also performed to investigate the cooccurrence of these genes in tumorigenesis.

**Results:**

We identified eight VS recurrence-related genes from the GSE141801 dataset. All of these genes were highly expressed in the VS recurrence samples. Four collagen family genes (*COL5A1*, *COL3A1*, *COL4A1*, and *COL15A1*) were further screened, and a model was constructed to predict the risk of recurrence of VS. Gene Ontology (GO) and Kyoto Encyclopedia of Genes and Genomes (KEGG) enrichment analyses revealed that these four collagen family genes play important roles in a variety of biological functions and cellular pathways. Pan-cancer analysis further revealed that the expression of these genes was significantly heterogeneous across immune phenotypes and significantly associated with immune infiltration. Finally, Neuropeptide Y (NPY) was found to be significantly and negatively correlated with the expression of *COL5A1*, *COL3A1*, and *COL4A1*.

**Conclusions:**

Four collagen family genes have been identified as possible predictors of recurrence after radiation therapy for VS. Pan-cancer analysis reveals potential associations between the pathogenesis of VS and other tumorigenic factors. The relevance of NPY to VS was also revealed for the first time.

## 1. Introduction

Vestibular schwannoma (VS) is a benign tumor that originates from the auditory nerve sheath and accounts for 8% to 10% of intracranial tumors with a similar incidence on the left and right sides, and occasionally bilateral [1]. VS is most common in adults, 30-50 years old; however, there is no significant gender difference. The main clinical manifestations are pontocerebellar horn syndrome and increased intracranial pressure. When the size of the tumor is small, patients will experience tinnitus, hearing loss, and vertigo on one side, and a few patients will become deaf after a longer period. As the tumor continues to grow, the patient will experience facial muscle twitching, reduced lacrimal secretion, facial numbness, reduced pain and touch, a weakened corneal reflex, and other symptoms [2]. Surgery is currently the main treatment option [3]; however there are many risks associated with surgical treatment, such as cerebrospinal fluid leakage with an incidence of around 2% to 30% [4].

Surgical treatment of VS no longer focuses solely on total removal of the tumor, but instead on protecting neurological function, reducing the incidence of postoperative complications, and improving the patient's post-operative quality of life. As a result, some of the newer VS treatments include microsurgery, stereotactic radiosurgery (SRS), fractionated stereotactic radiotherapy (FSRT), and targeted drug therapy [5–8]. The choice of different treatment modalities greatly impacts prognosis, functional preservation, and long-term quality of life. This has necessitated the medical staff that treats VS to grow from a single neurosurgeon to a multidisciplinary treatment team. The SRS and FSRT technologies are new technologies born out of this multidisciplinary collaboration. With the accumulation of long-term clinical treatment data and practical experience, the safety of SRS technology for treating VS has fewer side effects and a high tumor control rate [5–8]. In conclusion, radiotherapy shows great potential advantages as an alternative to surgery, taking into account patient comfort, quality of life, cost of treatment, and avoidance of potential surgical complications (i.e., meningitis, hemorrhage, cerebrospinal fluid leakage, hearing, and neurological collateral damage).

However, 34.7% of VS patients relapsed after SRS treatment [9]. Therefore, further research is still needed on the efficacy of radiotherapy for different types of VS. [10, 11] Several studies have examined the transcriptomic profile of different types of VS, but few have systematically explored the genes associated with SRS efficacy [12–15]. Indeed, if the molecular biological features associated with VS recurrence can be identified, more precise VS treatments can be achieved. The GSE141801 dataset from the Gene Expression Omnibus (GEO) database analyzes the transcriptomic profile of tumors between patients with VS who relapsed after radiation therapy alone and another group of patients who underwent direct surgery without radiation therapy [16].

Tumor recurrence after radiotherapy is closely related to vascular infiltration. Tumor recurrence areas have higher vascular and cell density, and vascular infiltration plays an important role in the development of tumors [17, 18]. The relationship between vascular infiltration and vestibular Schwannoma has been revealed in recent years [19, 20]. We speculate that excessive vascular infiltration may be associated with recurrence of VS after radiotherapy. Exploring angiogenic genes can help reveal the mechanism of VS recurrence.

Neuropeptide Y (NPY) is a 36 amino acid peptide that is widely distributed in the central and peripheral nervous systems. NPY infiltration is a manifestation of innervated tissues and cells [21]. Neuropeptides also have an effect on vascular development, and neuropeptides such as NPY are widely distributed in the perivascular area [22, 23]. Now, upregulation of NPY has been found to be associated with abnormal vascular function [24]. We speculate that NPY may have a potential relevance to the recurrence of vestibular schwannoma by regulating vascular-related function.

In this study, we used bioinformatics analysis to obtain genes associated with VS recurrence and studied important genes associated with angiogenesis among them and NPY. Pan-cancer analysis investigated the commonality of these genes in tumorigenesis.

## 2. Materials and Methods

### 2.1. Data Download and Preprocessing

We downloaded transcriptome microarray data and corresponding clinical data from the GSE141801 dataset for 67 patients with VS; of these, nine patients relapsed after radiation therapy and 58 patients were a first diagnosis. We transformed the microarray gene names according to the microarray platform file and then obtained the gene expression matrix. The angiogenesis-associated gene set was retrieved and downloaded from the MSigDB database (http://www.gsea-msigdb.org/gsea/msigdb/index.jsp). 226 angiogenesis-related genes were downloaded and collated from the MSigDB database. The 33 pan-cancer transcriptome expression data, immune subtypes, tumor microenvironment score data, and clinical information data from the Cancer Genome Atlas (TCGA) were downloaded from UCSC Xena (https://xenabrowser.net/datapages/).

### 2.2. Differentially Expressed Genes

The limma package performed batch correction of gene expression on intersample microarrays and tested for differences between the postradiotherapy relapse and nonradiotherapy groups. Differential genes were filtered by FDR < 0.05 and log_2_FC > 1. GO and KEGG performed a pathway enrichment analysis of up- and downregulated genes in the tumor tissue, respectively.

### 2.3. Angiogenesis-Related Genes

We performed intersection analysis between differential genes and the set of angiogenesis-related genes. We then obtained the angiogenesis-related differentially expressed genes (DEGs). Heat and volcano maps were used to demonstrate the gene expression and fold change of angiogenesis-related DEGs.

### 2.4. Logistic Regression Model Construction for Predicting Recurrence Rates after Radiation Therapy

Univariate and multifactorial logistic regression analyses were used for the analysis of angiogenesis-related DEGs and clinical characteristics. The filtering criterion of risk factors for recurrence after radiotherapy was *P* < 0.1, and risk factors were then screened for use in constructing logistic regression models. Next, we further constructed a nomogram to calculate the probability of recurrence after radiotherapy in VS patients for ease of use in the clinic.

### 2.5. Clinical Predictive Model Validation

The Caret package was used to split the entire dataset into training and test groups by 7 : 3. The model was trained in the training group and then validated in the test group. The receiver operating characteristic (ROC) curve and *C*-index were used to assess the predictive classification ability of the model in the training group, the overall cohort, and the test group. *C*-indices between 0.7 and 1.0 represented good predictive performance of the model. A calibration curve was also produced to assess the calibration of the model. Finally, decision curves were used to assess the net benefit at different probability thresholds and also to assess the clinical usability and safety of the nomogram and the model.

### 2.6. Pan-Cancer Analysis

We performed a pan-cancer analysis of the genes included in the model in the TCGA database. First, we performed differential gene expression analysis of the included genes in pan-cancerous and corresponding paracancerous tissues. Correlation with heat maps was used to demonstrate the relationship between incorporated gene expressions in pan-cancerous tissues. Cox proportional regression models divided tumor patients into the high- and low-expression groups by median gene expression, and the KM method was then used to perform survival curve mapping. Finally, the relationship between genes incorporated into the model, immune-related features, and tumor microenvironment scores were further analyzed.

### 2.7. Statistical Analysis

All statistics were plotted using R software (version 4.0.5). All statistical defaults were bilateral, while *P* < 0.05 was considered to be statistically significant. The ROCR package was used to plot ROC curves; the Hmisc package was used to calculate the *C*-index. The rms package was used for plotting the nomograms and calibration curves. The rmda package was used to plot the decision curve analysis (DCA) curves.

## 3. Results

### 3.1. Analysis of the Differences between the Recurrence Group and the First Diagnosis Group after Radiotherapy

**Figure 1:**
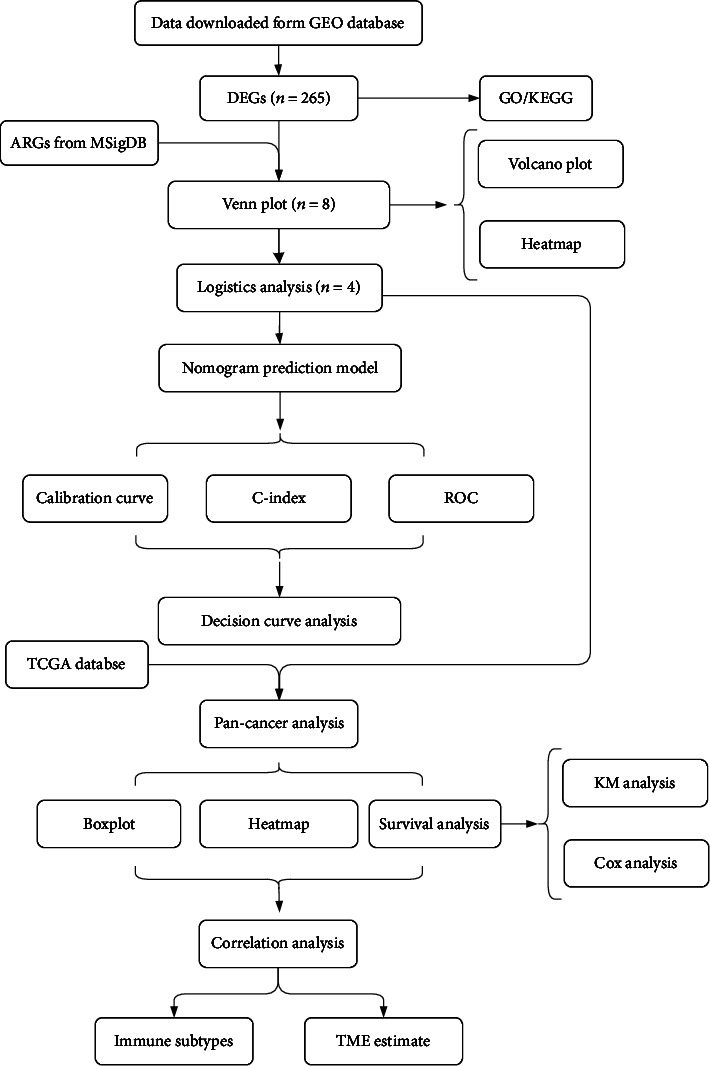
Flowchart of the analysis process. ARGS: angiogenesis-related genes; DEGs: differentially expressed genes; TME: tumor microenvironment.

The research flow chart is shown in Figure 1. The results of the differential analysis of gene expression in both groups of patients were saved in Supplementary table 1, and a total of 265 DEGs were obtained. GO and KEGG functional pathway analysis results are presented in a bar chart (Supplementary Figure 1), and Table 1 shows the top 10 up- and downregulated pathways in KEGG.

### 3.2. Angiogenesis-Related DEGs

**Figure 2:**
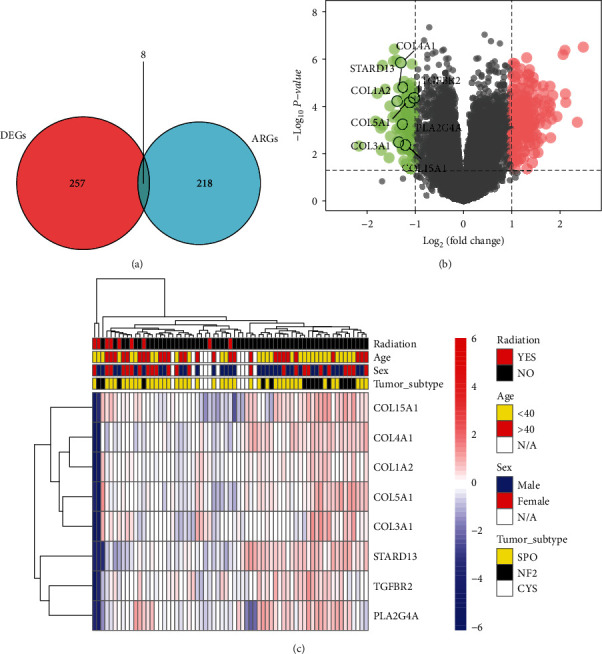
Results of differential and intersection analysis. (a) Venn diagram showing eight genes after taking intersection of DEGs and ARGs. (b) Volcano diagram showing differential and intersection genes. (c) Heat map showing expression of intersecting genes in tumor tissue and relationship to clinical traits.

Venn diagrams and volcano plots (Figures 2(a) and 2(b)) showed that the eight DEGs were also angiogenesis-related genes. All eight of these DEGs were highly expressed in the recurrence group after radiation therapy.  Table 2 shows the analysis of variance for these eight DEGs, and the heat map (Figure 2(c)) shows their expression of in tumor tissue and their relationship with clinical traits. These results suggest that the high expression of these eight DEGs is associated with recurrence after radiation therapy.

### 3.3. Single- and Multifactor Logistic Regression Analysis

All characteristics were included for single- and multifactor logistic regression analyses (Table 3). Seven genes (*COL4A1*, *STARD13*, *COL5A1*, *PLA2G4A*, *COL3A1*, *COL15A1*, and *TGFBR2*) were filtered for multivariate analysis at *P* < 0.1, and the final four collagen family genes (*COL5A1*, *COL3A1*, *COL4A1*, and *COL15A1*) were retained for further analysis.

### 3.4. Construction and Evaluation of the Logistics Regression Model

**Figure 3:**
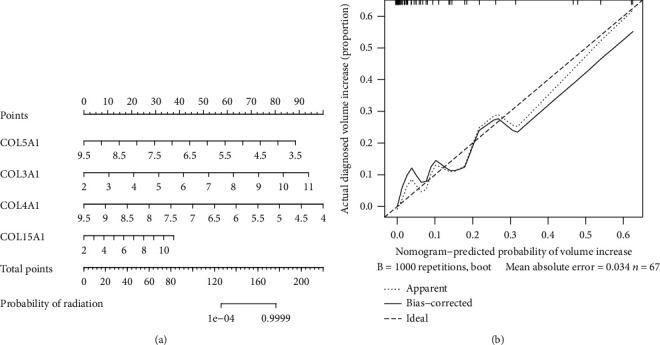
(a) Nomogram showing the column line graph prediction model for recurrence after radiotherapy. (b) Calibration graph showing the calibration of the prediction model.

**Figure 4:**
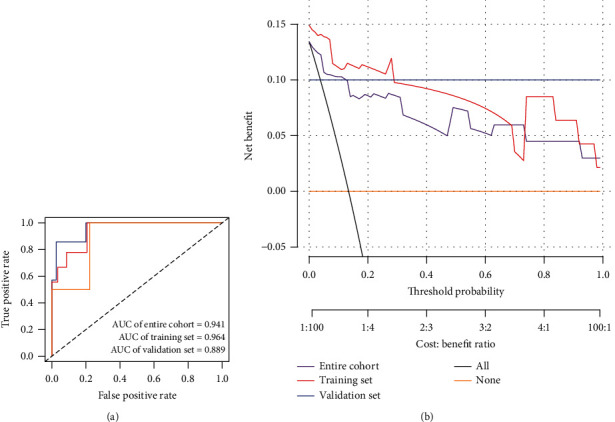
(a) ROC curves showing the classification and predictive efficacy of the predictive model. (b) DCA curves showing the range of clinical predictive safety.

*COL5A1*, *COL3A1*, *COL4A1*, and *COL15A1* were included for logistic regression model construction. The weights and statistical differences of the included factors in the constructed logistics regression model are shown in Table 4. A nomogram was used to calculate the likelihood of recurrence after radiotherapy according to a logistics regression model (Figure 3(a)), and a calibration graph evaluated the calibration of the fit of the model predictions and the actual classification (Figure 3(b)).  Figure 4(a) shows the ROC curves and area under the curve (AUC) values for the model in the training set, test set, and overall cohort, respectively (training set: 0.964, validation set: 0.889, and entire cohort: 0.941).  Table 5 shows the *C*-index for the three groups and ranges from 0.889-0.964, indicating that this model had good predictive classification efficacy. The DCA curve demonstrated that the model had a good range of reliability and safety in clinical prediction (Figure 4(b)). These results above show that the model has excellent predictive power. Therefore, the four collagen family genes were further screened by combining the clinical information provided from the database with the results of univariate and multivariate logistic analyses and were used to construct a prediction model for the risk of recurrence of VS.

### 3.5. Pan-Cancer Analysis

**Figure 5:**
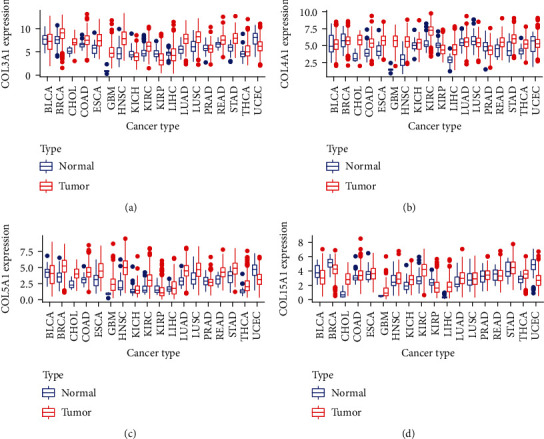
Box plot showing the expression of the four genes in the pan-cancerous tissue and its paracancerous tissues.

**Figure 6:**
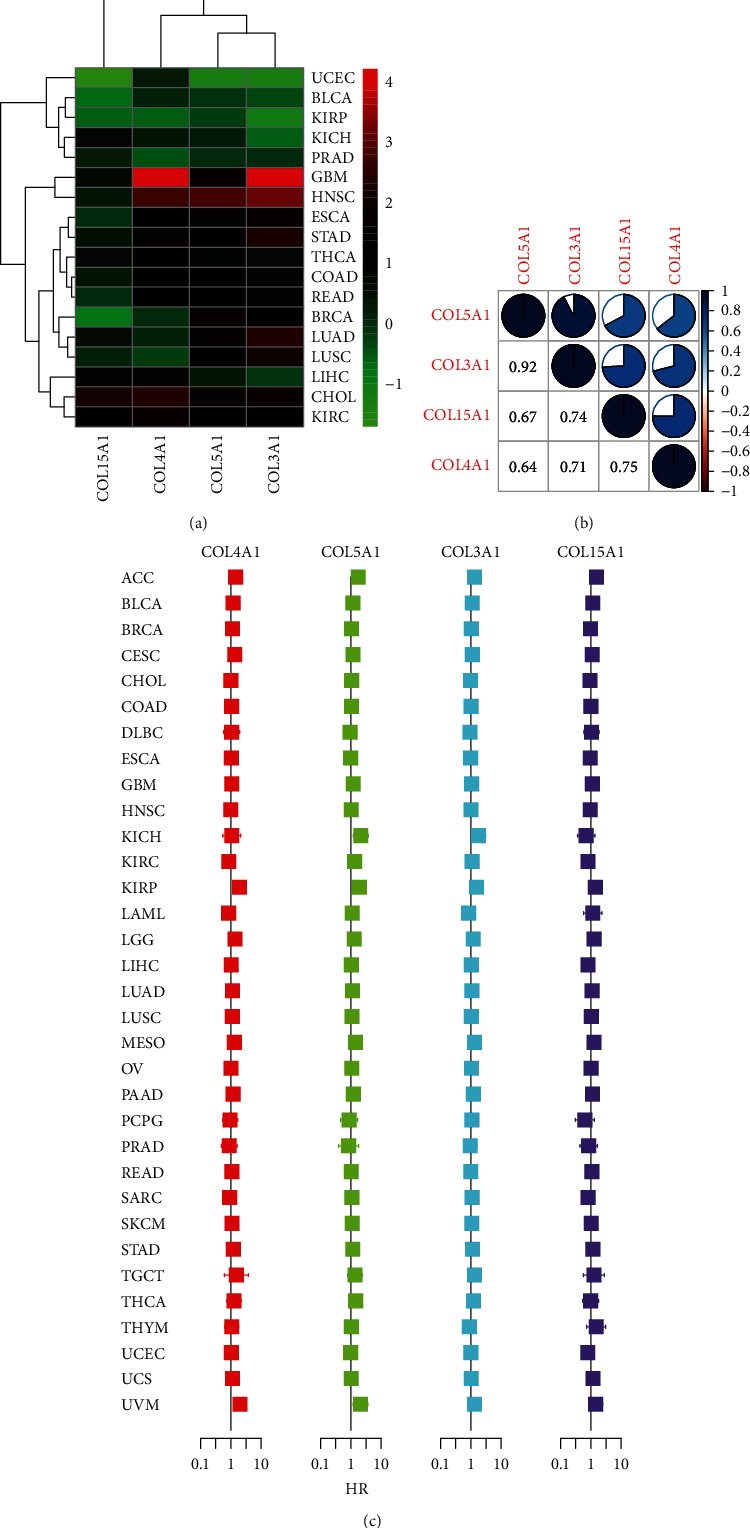
(a) Heat map showing the expression of the four genes in pan-cancerous tumor tissue. (b) Correlation heat map showing the correlation results of the expression of the four genes in pan-cancerous tissue. (c) Cox analysis showing the results of the four genes in pan-cancerous survival analysis. Intersection with the midline represents no statistical significance.

We further explored the expression of these four genes in pan-cancer and their role in the tumor microenvironment. Figures 5(a)–5(d) show that these four genes were relatively highly expressed in pan-cancerous tissues compared to their paracancerous counterparts.  Figure 6(a) shows how the expression of these four genes was relatively high in GBM, HNSC, STAD, LUAD, and CHOL and relatively low in UCEC, BLCA, KIRP, and PRAD, and Figure 6(b) shows the positive correlation between the expressions of these four genes in the pan-cancerous tissue.

### 3.6. Survival Analysis

**Figure 7:**
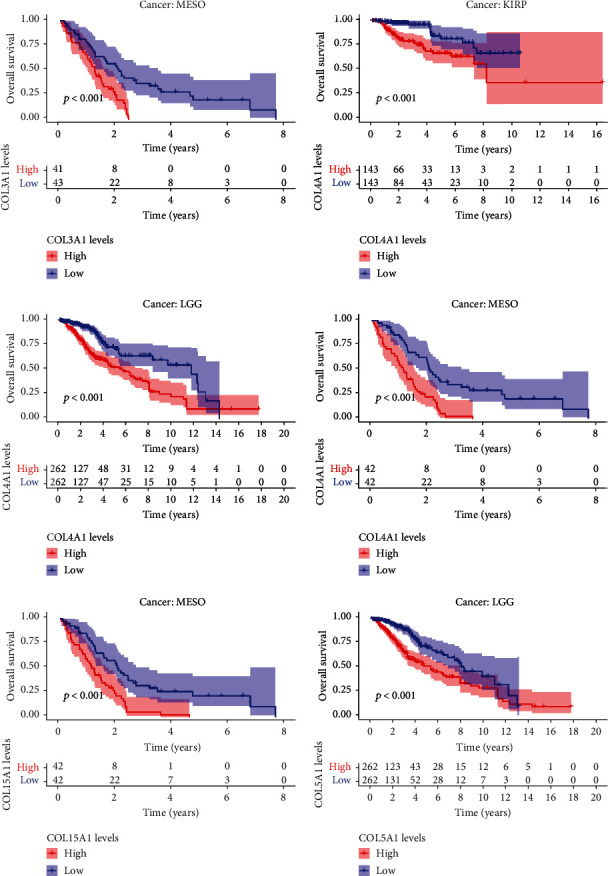
Survival curves showing the results of four genes in MESO, KIRP, and LGG (KM method).

We applied the KM method and Cox proportional regression models to the survival analysis of four genes in pan-cancer.  Figure 6(c) shows the results of applying cox regression analysis to the four genes in the pan-cancer. The HR and significance results of these four genes for pan-cancer were shown in Figure 6(c).  Figure 7 shows the statistically significant differences in the survival analysis of these four genes in MESO, KIRP, and LGG (*P* < 0.001).

### 3.7. Immune Subtypes and the Tumor Microenvironment

**Figure 8:**
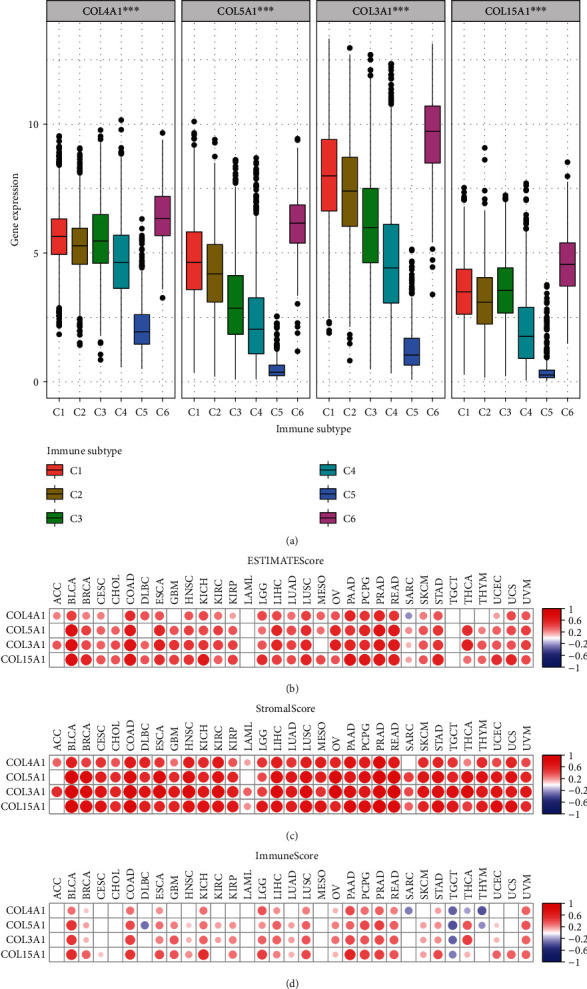
The results of the analysis of variance and correlation analysis demonstrate the relationship between the four genes and (a) the tumor immune subtype and (b–d) the tumor microenvironment score. Blanks in the heat map represent no statistically significant differences in correlation analysis.

We performed differential analysis and correlation analysis of these four genes and tumor immune subtypes with tumor microenvironment scores. In these 33 cancers, these four genes differed significantly in the six tumor immune subtypes (C1, C2, C3, C4, C5, and C6) (*P* < 0.05), Figure 8(a)). These four genes (*COL5A1*, *COL3A1*, *COL4A1*, and *COL15A1*) and the stromal, immune, and total scores in the tumor microenvironment were significantly correlated in most tumors (Figures 8(b)–8(d)).

### 3.8. Correlation of NPY with Collagen Family Genes and Vestibular Schwannoma Recurrence after Radiotherapy

**Figure 9:**
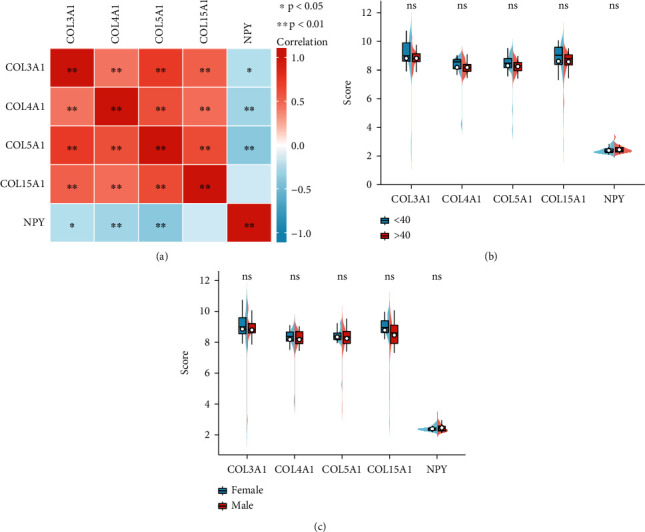
Correlation of NPY with collagen family genes and tumor recurrence after radiotherapy. Correlation analysis of NPY, COL3A1, COL4A1, COL5A1, and COL15A1 with each other (a). Differential expression of these genes (NPY, COL3A1, COL4A1, COL5A1, and COL15A1) in different ages and genders.

Four collagen family genes (COL3A1, COL4A1, COL5A1, and COL15A1) were significantly positively correlated with each other (Figure 9(a)). Low expression of COL4A1 and COL5A1 was associated with recurrence of vestibular schwannoma, while high expression of NPY was associated with recurrence of vestibular schwannoma (Table 6). In addition, these genes were not significantly associated with age and sex (Figures 9(b) and 9(c)). These results suggest that NPY is significantly associated with four collagen family genes (COL3A1, COL4A1, COL5A1, and COL15A1) and recurrence after radiotherapy for vestibular schwannoma.

## 4. Discussion

In this study, genes associated with VS recurrence were obtained using bioinformatics analysis. To investigate the role of angiogenic genes in this process, we obtained a collection of angiogenesis-related genes at MSigDB and intersected them with differentially expressed genes from the GSE141801 dataset. Eight genes were obtained for univariate and multifactorial logistic analyses, and four genes (*COL5A1, COL3A1, COL4A1*, and *COL15A1*) were screened. A column line graph prediction model was constructed by applying logistic regression to predict the recurrence status after radiation therapy. To further investigate the commonality of these genes in tumorigenesis, pan-cancer analysis was used to explore the role of these four target genes in tumor development. Finally, the relevance of NPY to vestibular schwannoma was also revealed for the first time.

We identified eight genes from the GSE141801 dataset that were highly expressed in the VS recurrence samples (including: *COL15A1*, *COL4A1*, *COL1A2*, *COL5A1*, *COL3A1*, *STARD13*, *TGFBR2*, and *PLA2G4A*). Four collagen family genes (*COL5A1*, *COL3A1*, *COL4A1*, and *COL15A1*) were further screened by combining the clinical information provided by the database with the results of univariate and multifactorial logistic analyses, and a prediction model for the risk of recurrence of VS was constructed accordingly. These four collagen family genes were found to be highly expressed in most tumor tissues. There was significant heterogeneity in the expression of these genes in different immunophenotypes. We assessed the association of these four collagen family genes (*COL5A1*, *COL3A1*, *COL4A1*, and *COL15A1*) with immune infiltration using three scoring systems (including: ESTIMATEScore, StromalScore, and StromalScore). With the exception of ACC, LAML, and SARC, all of these genes (*COL5A1*, *COL3A1*, *COL4A1*, and *COL15A1*) were found to be significantly associated with immune infiltration. KEGG and GO enrichment analyses revealed that these four collagen family genes played important roles in a variety of biological functions and cellular pathways. Furthermore, NPY was found significantly associated with four collagen family genes (COL3A1, COL4A1, COL5A1, and COL15A1) and recurrence after radiotherapy for VS.

M2-type macrophages in VS are associated with angiogenesis and tumor growth [25]. Collagen cleavage leads to increased macrophage adhesion and promotes macrophage infiltration. [26] The expression of three collagen family genes (COL5A1, COL3A1, and COL4A1) was negatively correlated with the expression of NPY, which was found to promote the migration of macrophages in collagen in vitro [27]. The crosstalk between collagen production and radiotherapy has been studied extensively [16, 17]. We have revealed an important function of these four collagen family genes (*COL5A1*, *COL3A1*, *COL4A1*, and *COL15A1*) in VS, and their high expression may be associated with VS radiotherapy recurrence. Mutations in *COL3A1* are associated with the development of mesothelioma. [28] High expression of *COL4A1* is associated with poor prognosis in renal papillary cell carcinoma [29]. In lower-grade glioma, *COL3A1*, *COL4A1*, and *COL5A1* are associated with patient prognosis and tumor progression [30, 31]. Also, the prognosis of mesothelioma is associated with *COL3A1*, *COL4A1*, and *COL15A1*. In addition, *COL3A1*, *COL4A1*, *COL5A1*, and *COL15A1* are associated with immune infiltration in head and neck squamous cell carcinoma, breast cancer, mesothelioma, and other tumors [32–35]. We performed differential analysis and correlation analysis of these four genes (*COL3A1*, *COL4A1*, *COL5A1*, and *COL15A1*) and tumor immune subtypes with tumor microenvironment scores. Our results are consistent with previous studies, showing that these four genes and the stromal scores, immune scores, and total scores in the tumor microenvironment are significantly correlated in most tumors. We used bioinformatics analysis to obtain genes associated with VS recurrence and vascularity, and the pan-cancer analysis allowed the commonality of these genes in tumorigenesis to be studied. Therefore, our database-based pan-cancer analysis suggests that these four collagen family genes (*COL5A1*, *COL3A1*, *COL4A1*, and *COL15A1*) have commonality with the progression of various tumors.

Low expression of COL4A1 and COL5A1 was associated with recurrence of vestibular schwannoma; while high expression of NPY was associated with recurrence of vestibular schwannoma. NPY was found to promote the migration of macrophages in collagen in vitro [27]. Recent studies have shown that these four collagen family genes (COL3A1, COL4A1, COL5A1, and COL15A1) are regulated by macrophages [27, 36, 37]. We speculate that NPY may influence VS angiogenesis by affecting macrophages to regulate the expression of COL5A1, COL3A1, and COL4A1. This hypothesis needs to be tested by further studies.

There are various methods of staging for VS. Among them, Koos grading method should be used in the future. According to the size of the tumor, VS can be classified into 4 grades. In grade 1, tumor is confined to the internal auditory tract; in grade 2, tumor invades the pontocerebellar horn, diameter ≤ 2 cm; in grade 3, tumor occupies the pontocerebellar horn pool without brainstem displacement, ≤3 cm; and in grade 4, huge tumor, >3 cm, with brainstem displacement. And the specific mechanisms by which these four collagen family genes are associated with recurrence after radiation therapy for VS remains unstudied. In the future, VS-related single-cell RNA-seq would validate our findings. Second, the association of these four collagen family genes with NPY has not been elucidated. Third, more cellular and animal experiments need to be performed to further explore the mechanisms involved. In addition, we have only focused on the expression abundance of these genes; consequently, gene polymorphisms also need to be explored. Third, all the data are from the database and we will need sufficient specimens from the clinic in the future to verify this conclusion. In addition, patients who have not relapsed after radiotherapy should be selected as controls versus those who have relapsed after radiotherapy, which will improve the scientific validity of future studies. Therefore, future cohort studies and controlled population-based pathology studies are necessary.

## 5. Conclusions

In this study, the expression of four angiogenesis-related collagen family genes (*COL5A1*, *COL3A1*, *COL4A1*, and *COL15A1*) was a predictor of recurrence after radiation therapy for VS. Pan-cancer analysis also revealed their potential correlation with the progression of other tumors, revealing an association between the pathogenesis of VS and other tumorigenic factors. And the relevance of NPY to VS was also revealed for the first time.

## Figures and Tables

**Table 1:** Up- and downregulated pathways in KEGG.

	Description	Adjust *P* value	geneID
Up	Nicotine addiction	*P* < 0.001	GRIA1/CACNA1A/GRIA4/GABRA1/GRIA2/GABRD
	Retrograde endocannabinoid signaling	*P* < 0.001	ADCY2/GNG4/GRIA1/CACNA1A/GRIA4/GABRA1/GRIA2/ADCY1/GABRD
	GABAergic synapse	*P* < 0.001	ADCY2/GNG4/CACNA1A/GABBR2/GABRA1/ADCY1/GABRD
	Morphine addiction	*P* < 0.001	ADCY2/GNG4/CACNA1A/GABBR2/GABRA1/ADCY1/GABRD
	Circadian entrainment	*P* < 0.001	ADCY2/GNG4/GRIA1/RYR3/GRIA4/GRIA2/ADCY1
	Glutamatergic synapse	*P* < 0.001	ADCY2/GNG4/GRIA1/CACNA1A/GRIA4/GRIA2/ADCY1
	cAMP signaling pathway	0.001	ADCY2/GRIA1/SOX9/ATP1B1/GABBR2/GRIA4/PPP1R1B/GRIA2/ADCY1
	Insulin secretion	0.001	ADCY2/ATP1B1/KCNN3/SNAP25/PCLO/ADCY1
	Adrenergic signaling in cardiomyocytes	0.003	ADCY2/PPP1R1A/ATP1B1/TNNC1/AGT/ADCY1/SCN4B
	Synaptic vesicle cycle	0.006	CACNA1A/SYT1/CPLX1/SNAP25/ATP6V1G2
Down	ECM-receptor interaction	*P* < 0.001	COL4A2/COL4A1/ITGA2/ITGB4/COL1A2/LAMB1/LAMA2/FREM2/FRAS1
	Protein digestion and absorption	*P* < 0.001	COL4A2/COL4A1/COL1A2/COL5A1/COL28A1/COL3A1/COL15A1
	Focal adhesion	*P* < 0.001	COL4A2/COL4A1/BIRC3/ITGA2/ITGB4/COL1A2/LAMB1/LAMA2
	Small cell lung cancer	*P* < 0.001	COL4A2/COL4A1/BIRC3/ITGA2/LAMB1/LAMA2
	Amoebiasis	*P* < 0.001	COL4A2/COL4A1/COL1A2/LAMB1/LAMA2/COL3A1
	Human papillomavirus infection	*P* < 0.001	COL4A2/COL4A1/ITGA2/ITGB4/COL1A2/LAMB1/LAMA2/FZD8
	AGE-RAGE signaling pathway in diabetic complications	*P* < 0.001	COL4A2/COL4A1/TGFBR2/COL1A2/COL3A1
	PI3K-Akt signaling pathway	*P* < 0.001	COL4A2/COL4A1/ITGA2/ITGB4/COL1A2/LAMB1/LAMA2/ERBB3
	Relaxin signaling pathway	0.002	COL4A2/COL4A1/TGFBR2/COL1A2/COL3A1
	Proteoglycans in cancer	0.012	ITGA2/RRAS/COL1A2/ERBB3/FZD8

**Table 2:** Eight different expression genes.

Id	logFC	*t*	*P* value	Adjust *P* value	*B*
COL4A1	-1.30	-5.30	1.42*E* − 06	1.00*E* − 03	5.11
STARD13	-1.25	-4.66	1.54*E* − 05	2.64*E* − 03	2.91
TGFBR2	-1.01	-4.38	4.34*E* − 05	3.81*E* − 03	1.97
COL1A2	-1.38	-4.29	5.95*E* − 05	4.18*E* − 03	1.68
COL5A1	-1.10	-4.25	6.69*E* − 05	4.40*E* − 03	1.57
PLA2G4A	-1.26	-3.62	5.72*E* − 04	1.15*E* − 02	-0.37
COL3A1	-1.34	-3.05	3.28*E* − 03	3.24*E* − 02	-1.93
COL15A1	-1.19	-2.97	4.12*E* − 03	3.73*E* − 02	-2.13

**Table 3:** Uni- and multilogistics regression analyses for recurrence after radiation.

Variables	Unilogistics regression			Multilogistics regression		
	*β*	Odds ratio (95% CI)	*P* value	*β*	Odds ratio (95% CI)	*P* value
COL4A1	-3.485	0.031 (0.001-0.309)	0.012	-6.812	0.001 (0-0.258)	0.065
STARD13	-1.355	0.258 (0.07-0.579)	0.008	0.613	1.847 (0.141-114.581)	0.670
TGFBR2	-2.171	0.114 (0.009-0.531)	0.052	-6.969	0.001 (0-1.695)	0.177
COL1A2	-1.773	0.17 (0.008-0.703)	0.198			
COL5A1	-1.682	0.186 (0.025-0.593)	0.043	-10.102	0 (0-0.066)	0.045
PLA2G4A	-0.870	0.419 (0.193-0.758)	0.010	-0.365	0.694 (0.046-9.363)	0.766
COL3A1	-0.551	0.577 (0.281-0.903)	0.037	4.460	86.455 (3.837-16515.578)	0.020
COL15A1	-0.627	0.534 (0.282-0.884)	0.025	3.490	32.775 (2.271-5105.412)	0.058
Tumor_subtype (CYS)						
NF2	0.731	2.077 (0.223-45.81)	0.553			
SPO	0.223	1.25 (0.171-25.518)	0.847			

Note: *β* is the regression coefficient.

**Table 4:** Prediction factors for recurrence after radiation.

Variables	Prediction model		
	*β*	Odds ratio (95% CI)	*P* value
(intercept)	48.356	1.00*E* + 21 (6.21*E* + 08 − 1.49*E* + 42)	0.011
COL5A1	-5.393	0.005 (0-0.429)	0.042
COL3A1	3.812	45.238 (3.515-1849.766)	0.014
COL4A1	-6.648	0.001 (0-0.068)	0.007
COL15A1	1.525	4.596 (0.933-42.261)	0.098

Note: *β* is the regression coefficient.

**Table 5:** *C*-index of the nomogram prediction model.

Dataset group	*C*-index of the prediction model	
	*C*-index	The *C*-index (95% CI)
Training set	0.964	0.908-1
Validation set	0.889	0.707-1
Entire cohort	0.941	0.878-1

**Table 6:** The basic characteristics of the patients.

Characteristic	First diagnosis	Relapsed	*P* value
*n*	58	9	
Sex, *n* (%)			0.888
	9 (13.4%)	1 (1.5%)	
Female	20 (29.9%)	4 (6%)	
Male	29 (43.3%)	4 (6%)	
Age, *n* (%)			1.000
	9 (13.4%)	1 (1.5%)	
<40 year	26 (38.8%)	4 (6%)	
>40 year	23 (34.3%)	4 (6%)	
COL3A1, median (IQR)	8.91 (8.61, 9.31)	8.92 (8.35, 9.48)	0.720
COL4A1, median (IQR)	8.4 (8.09, 8.68)	7.9 (7.44, 8.1)	<0.001
COL5A1, median (IQR)	8.42 (8.15, 8.71)	8.13 (7.66, 8.16)	0.009
COL15A1, median (IQR)	8.81 (8.24, 9.22)	8.56 (7.72, 8.66)	0.108
NPY, median (IQR)	2.38 (2.27, 2.52)	2.63 (2.51, 2.66)	0.005

## Data Availability

Readers may contact the corresponding author to obtain the original data if desired.

## Abbreviations

ACC: Adrenocortical carcinoma
BLCA: Bladder urothelial carcinoma
BRCA: Breast invasive carcinoma
CESC: Cervical squamous cell carcinoma
CHOL: Cholangiocarcinoma
COAD: Colon adenocarcinoma
DEGs: Differentially expressed genes
DLBC: Lymphoid neoplasm diffuse large B-cell lymphoma
ESCA: Esophageal carcinoma
FSRT: Fractionated stereotactic radiotherapy
GBM: Glioblastoma multiforme
GEO: Gene Expression Omnibus
GO: Gene Ontology
LGG: Brain lower-grade glioma
HNSC: Head and neck squamous cell carcinoma
KEGG: Kyoto Encyclopedia of Genes and Genomes
KICH: Kidney chromophobe
KIRC: Kidney renal clear cell carcinoma
KIRP: Kidney renal papillary cell carcinoma
KM: Kaplan-Meier
LAML: Acute myeloid leukemia
LIHC: Liver hepatocellular carcinoma
LUAD: Lung adenocarcinoma
LUSC: Lung squamous cell carcinoma
MESO: Mesothelioma
NPY: Neuropeptide Y
OV: Ovarian serous cystadenocarcinoma
PAAD: Pancreatic adenocarcinoma
PCPG: Pheochromocytoma and paraganglioma
PRAD: Prostate adenocarcinoma
READ: Rectum adenocarcinoma
ROC: Receiver operating characteristic
SARC: Sarcoma
SKCM: Skin cutaneous melanoma
SRS: Stereotactic radiosurgery
STAD: Stomach adenocarcinoma
TCGA: The Cancer Genome Atlas
TGCT: Testicular germ cell tumors
THCA: Thyroid carcinoma
THYM: Thymoma
UCEC: Uterine corpus endometrial carcinoma
UCS: Uterine carcinosarcoma
UVM: Uveal melanoma
VS: Vestibular schwannoma.

## Supplementary Materials

Supplementary Materials Supplementary Figure 1: GEO database differential analysis of GO and KEGG pathway enrichment analyses. Supplementary Table 1: the results of the differential analysis of gene expression in both groups of patients.
