# Supplementary material for: Collagen Family Genes Associated with Risk of Recurrence after Radiation Therapy for Vestibular Schwannoma and Pan-Cancer Analysis
Source: Dis Markers. 2021 Oct 13;2021:7897994. doi: 10.1155/2021/7897994 (PMC8528601; doi:10.1155/2021/7897994)
Supplement: Supplementary Materials — Supplementary Figure 1: GEO database differential analysis of GO and KEGG pathway enrichment analyses. Supplementary Table 1: the results of the differential analysis of gene expression in both groups of patients. [file 7897994.f1.zip › supplementary (1).docx]

# SUPPLEMENTARY FIGURE

#
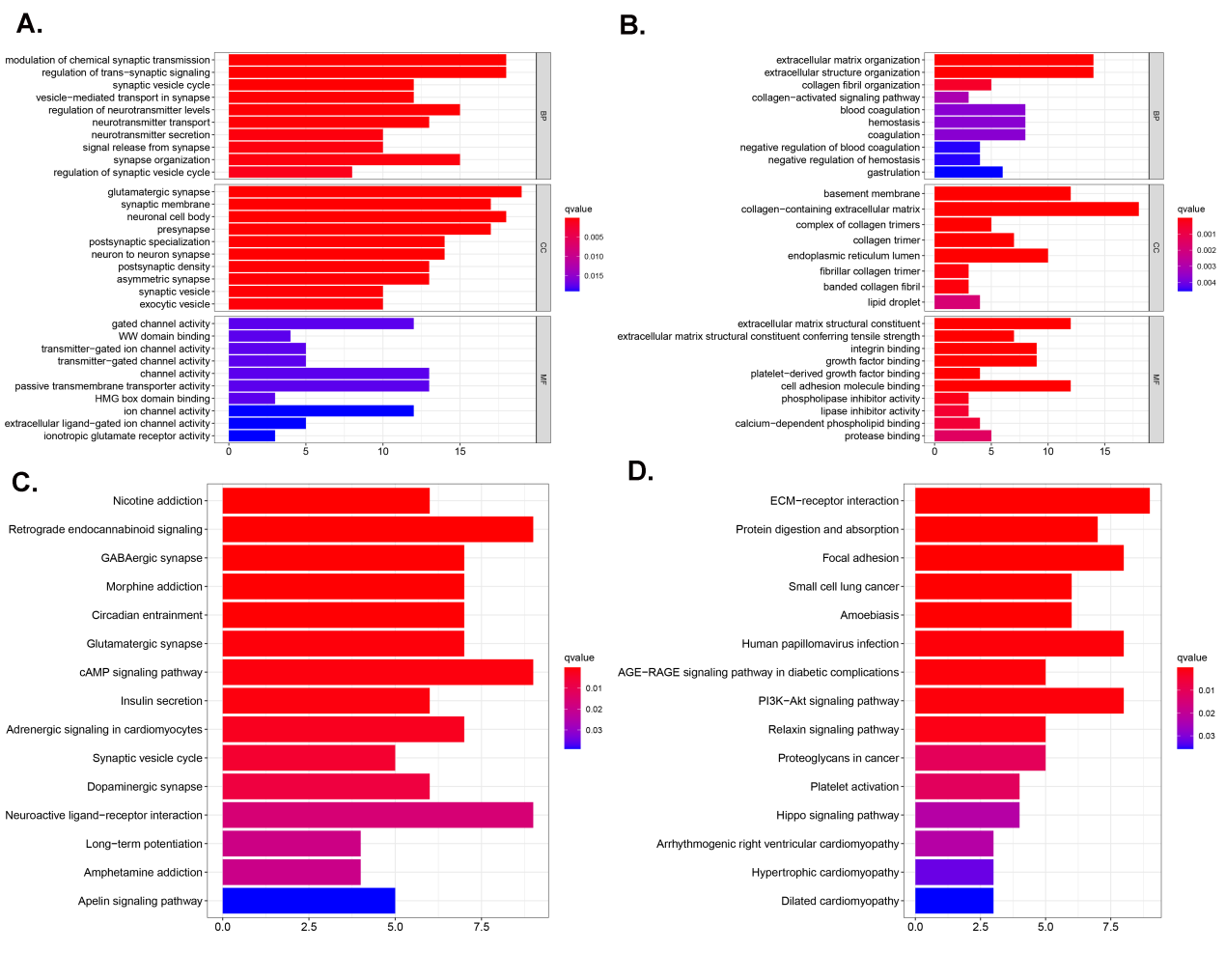


# **Supplementary Fig.1.** Demonstrated GEO database differential analysis of GO and KEGG pathway enrichment analysis. (A) GO-up, (B) GO-down, (C) KEGG-up, and (D) KEGG-down.
