# Supplementary material for: Collagen Family Genes Associated with Risk of Recurrence after Radiation Therapy for Vestibular Schwannoma and Pan-Cancer Analysis
Source: Dis Markers. 2021 Oct 13;2021:7897994. doi: 10.1155/2021/7897994 (PMC8528601; doi:10.1155/2021/7897994)
Supplement: Supplementary Materials — Supplementary Figure 1: GEO database differential analysis of GO and KEGG pathway enrichment analyses. Supplementary Table 1: the results of the differential analysis of gene expression in both groups of patients. [file 7897994.f1.zip › supplementary table 1.doc]

| Supplementary table 1: The results of the differential analysis of gene expression in both groups of patients | | | | | | |
| --- | --- | --- | --- | --- | --- | --- |
| id | logFC | AveExpr | t | P.Value | adj.P.Val | B |
| FBXL16 | 2.483028451 | 3.969573784 | 5.684149571 | 3.10E-07 | 0.00082682 | 6.501972292 |
| RNASE4 | -1.435522873 | 7.427935179 | -5.635334419 | 3.76E-07 | 0.00082682 | 6.324845076 |
| CEND1 | 2.102305874 | 3.621778216 | 5.604112569 | 4.25E-07 | 0.00082682 | 6.211807499 |
| SCG2 | 2.064157104 | 3.841666629 | 5.500417329 | 6.39E-07 | 0.000901492 | 5.837850151 |
| FJX1 | 1.308346422 | 4.931319199 | 5.419592763 | 8.75E-07 | 0.000916252 | 5.54800924 |
| SAMHD1 | -1.394021388 | 8.382097773 | -5.367984674 | 1.07E-06 | 0.000968121 | 5.36372788 |
| COL4A2 | -1.453970671 | 9.684728029 | -5.326690948 | 1.25E-06 | 0.001001381 | 5.216734731 |
| COL4A1 | -1.301848507 | 8.189850483 | -5.295341867 | 1.42E-06 | 0.001001381 | 5.105420008 |
| ADCY2 | 1.027775164 | 2.92935061 | 5.2796101 | 1.50E-06 | 0.001001381 | 5.049651588 |
| ASPHD2 | 1.054360697 | 4.006184504 | 5.271827879 | 1.55E-06 | 0.001001381 | 5.022086966 |
| LTBP4 | -1.069355187 | 6.487355132 | -5.26052855 | 1.62E-06 | 0.001001381 | 4.982092039 |
| BIRC3 | -1.682528514 | 6.386641042 | -5.229165966 | 1.83E-06 | 0.001001381 | 4.871252505 |
| ENAH | 1.0695686 | 4.945550452 | 5.219763582 | 1.89E-06 | 0.001001381 | 4.838072698 |
| LARP6 | 1.186581282 | 4.86732666 | 5.138703805 | 2.58E-06 | 0.001194636 | 4.552991277 |
| DNASE1L3 | 1.366569719 | 3.012538031 | 5.11715044 | 2.80E-06 | 0.001194636 | 4.47748774 |
| GNG4 | 1.128906924 | 2.934481751 | 5.009426941 | 4.22E-06 | 0.001382699 | 4.102071754 |
| ST8SIA1 | 1.350930486 | 2.812526846 | 5.004709316 | 4.29E-06 | 0.001383701 | 4.08570691 |
| IRX3 | 1.335686074 | 3.480678115 | 4.970691836 | 4.88E-06 | 0.001506423 | 3.967898408 |
| GRIA1 | 1.028478539 | 2.66042346 | 4.952440381 | 5.23E-06 | 0.001577973 | 3.904832135 |
| PAK3 | 1.067321457 | 3.314037684 | 4.934072932 | 5.60E-06 | 0.001609421 | 3.841466188 |
| CENPV | 1.442688297 | 3.271614944 | 4.906065953 | 6.22E-06 | 0.001666407 | 3.745042241 |
| IQCA1 | 1.227867704 | 3.496762609 | 4.866093028 | 7.23E-06 | 0.001807835 | 3.607840468 |
| ITGA2 | -1.228720598 | 6.330935232 | -4.814609141 | 8.75E-06 | 0.002106956 | 3.43186982 |
| SLITRK4 | 1.362471002 | 3.14195573 | 4.800343592 | 9.23E-06 | 0.002156145 | 3.383260727 |
| NMNAT2 | 1.07076312 | 3.378529834 | 4.787030997 | 9.70E-06 | 0.002169394 | 3.337958332 |
| TMEM140 | -1.083549861 | 7.218002995 | -4.761034886 | 1.07E-05 | 0.002259105 | 3.249661483 |
| RAB15 | 1.419767983 | 5.23229028 | 4.76021155 | 1.07E-05 | 0.002259105 | 3.246868624 |
| AHR | -1.115038316 | 8.32514763 | -4.707331763 | 1.30E-05 | 0.002381075 | 3.067967553 |
| TMEM132C | 1.498139595 | 3.607623347 | 4.698970733 | 1.34E-05 | 0.002408462 | 3.039767066 |
| BEX2 | 1.651893689 | 4.386870091 | 4.695595223 | 1.36E-05 | 0.002415768 | 3.028388725 |
| APBA2 | 1.792664113 | 3.094469921 | 4.686914979 | 1.40E-05 | 0.002471025 | 2.999146781 |
| STARD13 | -1.250909094 | 8.174409873 | -4.66103811 | 1.54E-05 | 0.002643843 | 2.912126571 |
| TMEM176B | -1.347547613 | 10.02739596 | -4.6405967 | 1.66E-05 | 0.002678152 | 2.843549056 |
| ZNF385D | 1.17776916 | 2.549962538 | 4.638834078 | 1.67E-05 | 0.002678152 | 2.837642585 |
| ITGB4 | -1.067623261 | 6.269978225 | -4.636231321 | 1.69E-05 | 0.002678152 | 2.828922848 |
| SLC7A11 | 1.261059248 | 3.944005248 | 4.626492776 | 1.75E-05 | 0.002732546 | 2.796317951 |
| TIAM2 | 1.021359213 | 3.115086507 | 4.612053018 | 1.85E-05 | 0.002787779 | 2.748034829 |
| ANXA3 | -1.625656271 | 8.043337778 | -4.608341585 | 1.87E-05 | 0.002801972 | 2.735636608 |
| FBXO41 | 1.053446841 | 3.582287362 | 4.591234533 | 1.99E-05 | 0.002808947 | 2.678553079 |
| GMPR | 1.349891583 | 3.99943871 | 4.588463122 | 2.01E-05 | 0.002808947 | 2.669315162 |
| RRAS | -1.037236374 | 8.493176404 | -4.543416373 | 2.37E-05 | 0.003123535 | 2.519549966 |
| FSTL3 | -1.072485607 | 6.863593087 | -4.538665 | 2.41E-05 | 0.003123535 | 2.503796295 |
| ARHGAP15 | -1.789790649 | 9.534397109 | -4.495631202 | 2.82E-05 | 0.003381645 | 2.361492522 |
| RNF182 | 1.66042065 | 2.465845415 | 4.492767776 | 2.85E-05 | 0.003381645 | 2.352048193 |
| AGXT2L1 | 2.112176302 | 3.095198377 | 4.481718376 | 2.96E-05 | 0.003381645 | 2.315633185 |
| PPP1R1A | 1.100721345 | 3.312096839 | 4.47849841 | 3.00E-05 | 0.003391487 | 2.305029923 |
| KAZ | 1.00050739 | 3.367356142 | 4.471395841 | 3.07E-05 | 0.003438399 | 2.281655167 |
| CNTNAP4 | 1.433206406 | 2.821428602 | 4.459713347 | 3.21E-05 | 0.003523778 | 2.243249241 |
| RYR3 | 1.226229055 | 2.588761625 | 4.45072179 | 3.31E-05 | 0.003557232 | 2.213724996 |
| FAM107A | 1.849852055 | 4.697422192 | 4.439444703 | 3.45E-05 | 0.00363143 | 2.176739696 |
| TMEM176A | -1.169889089 | 10.6812309 | -4.428396595 | 3.59E-05 | 0.00363143 | 2.140552633 |
| LRRC16A | 1.299686013 | 3.26963107 | 4.412633183 | 3.80E-05 | 0.003664612 | 2.089002453 |
| SNCAIP | 1.047055299 | 3.188997183 | 4.40672805 | 3.88E-05 | 0.003685657 | 2.069716006 |
| RARRES2 | -1.165139684 | 10.30946696 | -4.404235551 | 3.91E-05 | 0.003700139 | 2.061579452 |
| TGFBR2 | -1.011834812 | 8.039505014 | -4.375219328 | 4.34E-05 | 0.003806833 | 1.967036969 |
| LRRN3 | 1.289959243 | 5.117541364 | 4.359712536 | 4.58E-05 | 0.003823122 | 1.91664751 |
| ABCA8 | -1.263961851 | 6.917410422 | -4.352194179 | 4.71E-05 | 0.003875694 | 1.892250834 |
| AIFM3 | 1.207810223 | 2.876731634 | 4.33502991 | 5.00E-05 | 0.003977723 | 1.83663807 |
| RAMP1 | 1.552423032 | 4.987611225 | 4.327862233 | 5.13E-05 | 0.003994608 | 1.813449474 |
| CCDC136 | 1.206834767 | 3.270417659 | 4.321095837 | 5.26E-05 | 0.003994608 | 1.791578039 |
| CRYM | 1.707754654 | 2.863472902 | 4.3063104 | 5.54E-05 | 0.004088317 | 1.743850467 |
| MN1 | 1.293168647 | 3.640471222 | 4.301357651 | 5.64E-05 | 0.00409559 | 1.727882736 |
| LOC643008 | -1.019349022 | 6.236292744 | -4.297166973 | 5.72E-05 | 0.004105697 | 1.714379714 |
| COL1A2 | -1.376967334 | 9.451430738 | -4.286050911 | 5.95E-05 | 0.004182904 | 1.678596707 |
| FABP7 | 1.275325414 | 3.403201129 | 4.284920752 | 5.97E-05 | 0.004182904 | 1.674961509 |
| DTNA | 1.012689054 | 3.736211141 | 4.268407686 | 6.33E-05 | 0.004270011 | 1.6219064 |
| CELF5 | 1.532136135 | 2.471071036 | 4.259030723 | 6.54E-05 | 0.004329628 | 1.591828972 |
| COL5A1 | -1.104191862 | 8.26947363 | -4.252870257 | 6.69E-05 | 0.004400644 | 1.572088533 |
| TCEAL2 | 2.120380574 | 4.075217568 | 4.249584979 | 6.77E-05 | 0.004405346 | 1.561567701 |
| CRMP1 | 1.148214669 | 3.575790683 | 4.24812185 | 6.80E-05 | 0.004405346 | 1.556883596 |
| NRXN2 | 1.781649716 | 3.739337724 | 4.247191053 | 6.82E-05 | 0.004405346 | 1.553904177 |
| SRRM3 | 1.187217251 | 2.501565956 | 4.245378304 | 6.87E-05 | 0.004405346 | 1.548102721 |
| PPM1H | 1.409835506 | 3.751979236 | 4.241639796 | 6.96E-05 | 0.004424589 | 1.536142456 |
| TCEAL5 | 1.492390336 | 3.336876787 | 4.235283345 | 7.11E-05 | 0.004439506 | 1.51582022 |
| BBOX1 | 1.307883161 | 2.523660579 | 4.22606216 | 7.35E-05 | 0.00444978 | 1.486369146 |
| TCEAL6 | 1.03383937 | 5.59932773 | 4.206671952 | 7.86E-05 | 0.004537351 | 1.424556233 |
| SOX9 | 1.190613388 | 3.893693266 | 4.203440382 | 7.95E-05 | 0.004537351 | 1.414269914 |
| OLFML2B | -1.138950236 | 7.853058374 | -4.199821991 | 8.05E-05 | 0.004537351 | 1.402757562 |
| KNDC1 | 1.150631181 | 2.766848443 | 4.198777142 | 8.08E-05 | 0.004537351 | 1.399434279 |
| CTNND2 | 1.666319453 | 3.727720897 | 4.196897293 | 8.14E-05 | 0.004542117 | 1.393456334 |
| LAMB1 | -1.019194662 | 7.807701935 | -4.17691926 | 8.72E-05 | 0.004631249 | 1.330018795 |
| CDC42EP5 | -1.227353499 | 5.73915536 | -4.173620625 | 8.82E-05 | 0.004648468 | 1.319560814 |
| OTX2 | 1.03885175 | 2.255238961 | 4.171143935 | 8.90E-05 | 0.004663511 | 1.311711787 |
| SLAIN1 | 1.279060308 | 2.592845898 | 4.169533568 | 8.95E-05 | 0.004663511 | 1.306609685 |
| PVALB | 1.801206595 | 2.960832753 | 4.166222254 | 9.05E-05 | 0.004701383 | 1.296122 |
| GAS2L3 | -1.527917435 | 9.049646125 | -4.14977242 | 9.59E-05 | 0.004836014 | 1.244091579 |
| IL17D | 1.042417556 | 4.680086642 | 4.145483294 | 9.73E-05 | 0.004842046 | 1.230544383 |
| LRRC3B | 1.231698244 | 2.702065282 | 4.130311533 | 0.00010257 | 0.004925426 | 1.18268845 |
| ZFPM2 | 1.334380459 | 3.191605971 | 4.125155328 | 0.000104418 | 0.004951637 | 1.166447165 |
| ATP1B1 | 1.169928657 | 6.365400665 | 4.121783252 | 0.000105643 | 0.004972563 | 1.155831899 |
| TMOD1 | 1.114131241 | 4.451614818 | 4.117498448 | 0.000107221 | 0.005009213 | 1.142350554 |
| ARSE | -1.03434052 | 5.909002553 | -4.093112154 | 0.000116641 | 0.005270597 | 1.065777106 |
| IRX5 | 1.014732211 | 2.701789311 | 4.086554571 | 0.000119308 | 0.00532571 | 1.045230937 |
| VGLL3 | -1.191273001 | 6.147633586 | -4.084767164 | 0.000120044 | 0.005336641 | 1.03963395 |
| RTN1 | 1.686470602 | 5.471395778 | 4.069305458 | 0.000126604 | 0.005516213 | 0.991277316 |
| DARC | 1.012905516 | 5.363552263 | 4.058783962 | 0.000131264 | 0.005571709 | 0.958432221 |
| CCDC80 | 1.422963621 | 3.986669096 | 4.053794889 | 0.000133532 | 0.005617778 | 0.942875094 |
| TJP2 | 1.124767027 | 4.915690972 | 4.051860194 | 0.000134421 | 0.005630066 | 0.936845259 |
| MYBPC1 | 1.165713343 | 2.709213545 | 4.048324675 | 0.000136061 | 0.005648436 | 0.925830507 |
| LIX1 | 1.067968409 | 2.457518785 | 4.046380164 | 0.000136971 | 0.005648436 | 0.919774867 |
| CACNA1A | 1.73079987 | 5.622719351 | 4.044631926 | 0.000137794 | 0.005658826 | 0.914331919 |
| RGN | 1.089144131 | 3.554373711 | 4.023982458 | 0.000147885 | 0.00587093 | 0.85014654 |
| PAPPA | -1.193689041 | 6.225435047 | -4.006931424 | 0.00015675 | 0.006043376 | 0.797292249 |
| TAGLN3 | 1.768484027 | 2.840144476 | 4.003553489 | 0.000158566 | 0.006043376 | 0.786837177 |
| NTSR2 | 1.374710053 | 2.826290037 | 3.993719377 | 0.000163968 | 0.006211196 | 0.756429319 |
| TTC9B | 1.058008714 | 2.605429182 | 3.991593803 | 0.000165159 | 0.006231476 | 0.749862711 |
| NELL2 | 1.053839019 | 2.871955402 | 3.987887264 | 0.000167255 | 0.006255601 | 0.738416949 |
| TNNC1 | 1.743219416 | 3.521849096 | 3.986313746 | 0.000168153 | 0.006255601 | 0.733559851 |
| GABBR2 | 1.784728625 | 2.921405589 | 3.982240032 | 0.000170499 | 0.006255601 | 0.720990509 |
| SLC25A18 | 1.562560022 | 3.577812818 | 3.981296937 | 0.000171046 | 0.006255601 | 0.718081707 |
| SGK223 | 1.0207495 | 7.704704513 | 3.954183321 | 0.000187529 | 0.006628356 | 0.634630964 |
| ELAVL3 | 1.322452323 | 2.768284841 | 3.949854448 | 0.000190298 | 0.006676591 | 0.621339149 |
| NOL4 | 1.057144356 | 2.396058559 | 3.947395726 | 0.000191889 | 0.006694548 | 0.613793538 |
| LAMA2 | -1.229865376 | 9.723712075 | -3.94053722 | 0.000196392 | 0.006777846 | 0.592760306 |
| PRSS23 | -1.17083389 | 9.436677893 | -3.934217016 | 0.000200632 | 0.006874269 | 0.573397437 |
| ZIC2 | 1.379267578 | 3.64172738 | 3.926038857 | 0.000206249 | 0.006988881 | 0.548370328 |
| LY6H | 1.089677258 | 2.706655425 | 3.923698481 | 0.000207884 | 0.007006458 | 0.541214022 |
| LGI1 | 1.444573538 | 3.717837751 | 3.920107158 | 0.000210417 | 0.007058962 | 0.530237645 |
| BEGAIN | 1.129747234 | 4.127176773 | 3.919996949 | 0.000210495 | 0.007058962 | 0.529900904 |
| FAM155A | 1.331470082 | 3.386104091 | 3.916935291 | 0.000212679 | 0.007083843 | 0.520548349 |
| ANXA2P2 | -1.040355244 | 11.86603305 | -3.902309052 | 0.000223415 | 0.007262317 | 0.475930346 |
| H2AFJ | -1.419977795 | 5.736616404 | -3.890765849 | 0.000232253 | 0.007397853 | 0.440789109 |
| PRKCDBP | -1.336995692 | 8.467490268 | -3.889710969 | 0.000233077 | 0.007401431 | 0.437580886 |
| CADPS | 1.287109469 | 2.50408262 | 3.886989147 | 0.000235216 | 0.00744239 | 0.429305418 |
| ELAVL2 | 1.2834799 | 2.53261662 | 3.883888636 | 0.000237677 | 0.007478364 | 0.419882899 |
| BCAN | 1.337065298 | 2.540429268 | 3.881221082 | 0.000239813 | 0.007501352 | 0.41177983 |
| MOBP | 1.066789934 | 2.542453812 | 3.873979194 | 0.000245706 | 0.007597968 | 0.38979879 |
| C14orf139 | -1.02372254 | 9.413009118 | -3.867972626 | 0.000250699 | 0.007657084 | 0.371586398 |
| PRKCZ | 1.275635528 | 4.112443303 | 3.857865651 | 0.000259321 | 0.007799759 | 0.340980499 |
| FGF14 | 1.000092536 | 2.438043667 | 3.857029505 | 0.000260047 | 0.007799759 | 0.338450695 |
| NID2 | -1.256467837 | 7.835893832 | -3.855693583 | 0.00026121 | 0.007811047 | 0.334409494 |
| TMEM108 | 1.099528111 | 2.567064291 | 3.854406671 | 0.000262336 | 0.007831374 | 0.330517365 |
| SPOCK1 | 1.936992251 | 5.641335095 | 3.846153065 | 0.000269668 | 0.007987542 | 0.305574298 |
| AGT | 1.677370635 | 4.151513504 | 3.833888892 | 0.000280926 | 0.008106342 | 0.268572104 |
| C8orf84 | -1.465739171 | 7.872686181 | -3.825740838 | 0.000288652 | 0.008254155 | 0.244029187 |
| FAM123A | 1.601117472 | 2.946031807 | 3.816227099 | 0.000297932 | 0.008396198 | 0.215413776 |
| KCND2 | 1.22809531 | 2.618398494 | 3.813462759 | 0.000300681 | 0.008441119 | 0.207107522 |
| INSM1 | 1.012450597 | 2.68132679 | 3.806148745 | 0.000308073 | 0.008456586 | 0.185148589 |
| AQP4 | 1.198636578 | 2.566025523 | 3.806041298 | 0.000308183 | 0.008456586 | 0.184826199 |
| PRRX1 | 1.329637179 | 4.535657963 | 3.802092305 | 0.000312248 | 0.008506742 | 0.172981262 |
| SFRP2 | 1.592061951 | 2.921277479 | 3.782750895 | 0.000332907 | 0.008903754 | 0.115078396 |
| RAB3IP | 1.085351131 | 3.894205928 | 3.776141137 | 0.000340263 | 0.008968929 | 0.095333149 |
| PRG4 | 1.066284501 | 2.729305909 | 3.774293168 | 0.000342347 | 0.008968929 | 0.089816626 |
| STMN2 | 1.828491491 | 2.958468503 | 3.769428363 | 0.000347892 | 0.009017743 | 0.075302438 |
| ANXA2 | -1.038064521 | 10.44274516 | -3.769267539 | 0.000348077 | 0.009017743 | 0.074822818 |
| CA10 | 1.136126773 | 2.466992452 | 3.76175468 | 0.000356813 | 0.009107594 | 0.052431964 |
| FREM2 | -1.282160002 | 4.089939831 | -3.760578892 | 0.000358199 | 0.009130719 | 0.048930275 |
| FRAS1 | -1.263195793 | 6.395363738 | -3.730367361 | 0.000395633 | 0.009632968 | -0.04080633 |
| SORCS1 | -1.21412128 | 7.879185789 | -3.72631392 | 0.000400931 | 0.009699867 | -0.052811121 |
| NEURL1B | -1.043926132 | 7.929669758 | -3.722592642 | 0.000405854 | 0.009711438 | -0.063824837 |
| VSTM2B | 1.072625759 | 2.481358407 | 3.721437802 | 0.000407394 | 0.009711438 | -0.06724134 |
| SYNGR3 | 1.010696534 | 2.771484513 | 3.718889752 | 0.00041081 | 0.009722188 | -0.074777152 |
| PHGDH | 1.081089396 | 5.937115652 | 3.718058201 | 0.000411931 | 0.009730792 | -0.077235737 |
| COL28A1 | -1.827458053 | 6.293255238 | -3.712590445 | 0.000419375 | 0.009845483 | -0.093393069 |
| ABCA6 | -1.304781573 | 6.522482372 | -3.711094695 | 0.000421434 | 0.009869442 | -0.097810389 |
| FIBIN | 1.419739322 | 4.422826857 | 3.698318788 | 0.000439415 | 0.010103884 | -0.135494283 |
| CPE | 1.459934576 | 6.031248603 | 3.694923887 | 0.000444315 | 0.010167371 | -0.145493864 |
| PAX6 | 1.118070121 | 2.644337737 | 3.691279989 | 0.000449632 | 0.010223388 | -0.156220287 |
| PLCXD3 | 1.282352525 | 2.677241736 | 3.687671331 | 0.000454957 | 0.01025055 | -0.166836262 |
| C1orf61 | 2.358805882 | 3.179940273 | 3.679189517 | 0.000467712 | 0.01042384 | -0.191761749 |
| PHACTR3 | 1.296912786 | 2.777990764 | 3.679164268 | 0.000467751 | 0.01042384 | -0.191835894 |
| C2orf82 | 1.101208617 | 2.972159607 | 3.67891047 | 0.000468137 | 0.01042384 | -0.192581154 |
| DSCAML1 | 1.291558001 | 2.633324971 | 3.667834221 | 0.000485323 | 0.010632374 | -0.225073469 |
| C15orf59 | 1.325527819 | 5.222959516 | 3.664568687 | 0.000490503 | 0.010658227 | -0.234640849 |
| ST6GALNAC2 | -1.770857043 | 9.505603631 | -3.65955496 | 0.00049856 | 0.010773423 | -0.24931934 |
| PRICKLE1 | 1.123906641 | 5.319815166 | 3.65413819 | 0.000507406 | 0.01092732 | -0.265163145 |
| SYT1 | 1.570423175 | 2.762285686 | 3.648549366 | 0.000516689 | 0.011039727 | -0.28149421 |
| RALYL | 1.228538995 | 2.934376921 | 3.642400317 | 0.00052709 | 0.011119183 | -0.299443509 |
| OLIG1 | 1.710811502 | 2.669729279 | 3.626289341 | 0.000555295 | 0.01141567 | -0.346378332 |
| C10orf10 | -1.281755614 | 8.301854493 | -3.623179064 | 0.000560903 | 0.011438865 | -0.355423588 |
| FLNC | 1.080190518 | 6.115064153 | 3.622995648 | 0.000561236 | 0.011438865 | -0.355956838 |
| BMP6 | -1.220928861 | 5.710993043 | -3.621621683 | 0.000563732 | 0.011465159 | -0.35995083 |
| PLA2G4A | -1.262400486 | 6.614060193 | -3.616925176 | 0.000572344 | 0.011527633 | -0.373595655 |
| FNDC5 | 1.117884919 | 3.577175751 | 3.616642584 | 0.000572866 | 0.011527633 | -0.374416304 |
| OLIG2 | 1.450208888 | 2.850135389 | 3.615312988 | 0.000575328 | 0.011540555 | -0.378276888 |
| AVPI1 | -1.066403347 | 8.937002402 | -3.610626444 | 0.00058409 | 0.011618257 | -0.39187721 |
| BSN | 1.281029758 | 3.3062743 | 3.606245378 | 0.000592395 | 0.011709962 | -0.404580552 |
| ZIC1 | 1.050671583 | 2.607937303 | 3.601373435 | 0.000601763 | 0.011821401 | -0.418695333 |
| GPR37 | 1.103413562 | 2.546425082 | 3.598131381 | 0.000608074 | 0.011927458 | -0.428081109 |
| ERBB3 | -1.209279725 | 7.854448322 | -3.591826269 | 0.00062053 | 0.012097531 | -0.446318509 |
| THSD4 | 1.410581717 | 4.002239371 | 3.583467063 | 0.000637417 | 0.012330753 | -0.470464782 |
| COL22A1 | 1.082377023 | 3.034498848 | 3.575611676 | 0.000653685 | 0.012505507 | -0.493121814 |
| SLC16A12 | -1.679229804 | 8.547848199 | -3.575184022 | 0.000654582 | 0.012510079 | -0.494354338 |
| APLNR | 1.403130903 | 3.564585746 | 3.574609118 | 0.000655789 | 0.012510353 | -0.496011093 |
| IL17RB | 1.035601816 | 2.972613535 | 3.573941396 | 0.000657194 | 0.012522254 | -0.497935104 |
| TIMP4 | 1.399431499 | 4.083243001 | 3.570777427 | 0.000663891 | 0.012574264 | -0.50704871 |
| BAALC | 1.375792039 | 6.48825812 | 3.557820207 | 0.000692 | 0.012875811 | -0.544315212 |
| CDO1 | 1.348580598 | 5.375989467 | 3.541617407 | 0.000728743 | 0.013299204 | -0.590789452 |
| DOCK3 | 1.047944327 | 4.696052632 | 3.531926698 | 0.000751595 | 0.01354724 | -0.618517456 |
| C12orf56 | -1.284518254 | 6.366737405 | -3.524955369 | 0.000768454 | 0.013727231 | -0.638433049 |
| MFAP5 | 1.15658098 | 3.078559458 | 3.515261217 | 0.000792495 | 0.014016419 | -0.666083282 |
| ASPA | -1.21331754 | 7.741574017 | -3.515019326 | 0.000793104 | 0.014016419 | -0.666772566 |
| C8orf4 | -1.098160462 | 8.803625292 | -3.510492156 | 0.000804582 | 0.014178457 | -0.679667103 |
| GRIA4 | 1.359354708 | 2.593067929 | 3.507452019 | 0.000812379 | 0.014260943 | -0.688319915 |
| KCNN3 | 1.067703185 | 3.096225652 | 3.50272388 | 0.000824646 | 0.014376131 | -0.701767074 |
| GFRA3 | -1.718842539 | 9.122686168 | -3.501467207 | 0.000827936 | 0.014404427 | -0.705339083 |
| CASKIN1 | 1.100613625 | 3.276328134 | 3.492940553 | 0.00085059 | 0.014637852 | -0.729552686 |
| RDH10 | -1.195188296 | 8.394932794 | -3.48348918 | 0.000876388 | 0.014762865 | -0.756345638 |
| LGI4 | -1.332326073 | 9.963607593 | -3.482892974 | 0.00087804 | 0.014762865 | -0.758034127 |
| KSR2 | 1.036970176 | 2.690711112 | 3.473946548 | 0.000903185 | 0.015000084 | -0.783347401 |
| CDH19 | -1.386219436 | 8.237641548 | -3.471350021 | 0.00091061 | 0.015000787 | -0.790685819 |
| SCN7A | -1.155566945 | 5.889070392 | -3.449168776 | 0.000976437 | 0.015508832 | -0.853223228 |
| GABRA1 | 1.163096858 | 2.436711977 | 3.447639584 | 0.000981137 | 0.015508832 | -0.857524535 |
| SEL1L3 | 1.114615767 | 5.93725184 | 3.417518517 | 0.001078204 | 0.01626419 | -0.941982709 |
| ODZ2 | 1.222479969 | 3.664361087 | 3.406853582 | 0.001114699 | 0.016661223 | -0.971764675 |
| ALDH1A1 | -1.089449822 | 11.04976022 | -3.379339357 | 0.001214318 | 0.017529055 | -1.048301591 |
| CPLX1 | 1.322779001 | 3.723163876 | 3.374535269 | 0.001232551 | 0.017675865 | -1.061621144 |
| TMEM163 | 1.446618929 | 4.85133985 | 3.370963478 | 0.001246275 | 0.017778823 | -1.07151557 |
| CTNNAL1 | -1.114032069 | 9.951858973 | -3.3706533 | 0.001247474 | 0.017782581 | -1.07237447 |
| FZD8 | -1.078069615 | 7.538413052 | -3.361798906 | 0.001282151 | 0.018046918 | -1.096869558 |
| INSC | -1.09176828 | 6.308832266 | -3.351626493 | 0.001323113 | 0.018417854 | -1.12495546 |
| MIA | -1.316007516 | 9.508064261 | -3.333194679 | 0.001400496 | 0.019145812 | -1.17569396 |
| NCAN | 1.135916587 | 2.544447549 | 3.330693384 | 0.00141132 | 0.019252265 | -1.18256435 |
| HSPB8 | 1.011594261 | 3.880133792 | 3.311378404 | 0.001497599 | 0.019914927 | -1.235495458 |
| DNER | 1.49983225 | 3.322783036 | 3.303864125 | 0.001532489 | 0.020233418 | -1.256029179 |
| LOC157503 | -1.215845791 | 7.330967464 | -3.301702372 | 0.001542667 | 0.020328688 | -1.261930351 |
| RAB26 | 1.279648572 | 2.609441191 | 3.295783908 | 0.001570859 | 0.020558469 | -1.278072677 |
| NTRK3 | 1.010517291 | 4.267221806 | 3.282940801 | 0.001633713 | 0.021119435 | -1.313031191 |
| PROS1 | -1.086571471 | 9.096242909 | -3.278106051 | 0.001657982 | 0.021317226 | -1.326166177 |
| SNCB | 1.025574091 | 2.585710931 | 3.276707307 | 0.001665066 | 0.021363074 | -1.329963707 |
| SNAP25 | 1.039055855 | 2.622474592 | 3.272472192 | 0.001686689 | 0.021526226 | -1.341454845 |
| CSPG5 | 1.102140771 | 5.476199925 | 3.268639729 | 0.001706484 | 0.021687867 | -1.351844373 |
| SCRG1 | 1.417397354 | 5.192614149 | 3.255408336 | 0.001776516 | 0.022194422 | -1.387647146 |
| NKAIN4 | 1.339214022 | 3.590654582 | 3.250940286 | 0.001800769 | 0.022381319 | -1.399713855 |
| SERPINA5 | -1.518092966 | 8.692027033 | -3.246188389 | 0.001826903 | 0.022558697 | -1.412534167 |
| BAMBI | 1.28315667 | 5.175438015 | 3.245744389 | 0.001829363 | 0.022574415 | -1.413731365 |
| CDK5R1 | 1.000795492 | 4.212370195 | 3.243982076 | 0.001839158 | 0.022635703 | -1.418482103 |
| PCLO | 1.003463509 | 2.608944162 | 3.243531295 | 0.001841672 | 0.022638158 | -1.419696996 |
| CRABP1 | 1.323369025 | 3.543972113 | 3.239160905 | 0.001866207 | 0.022807066 | -1.431469306 |
| C16orf89 | 1.344051542 | 5.917469823 | 3.214533734 | 0.002010301 | 0.024012487 | -1.497593651 |
| VSNL1 | 1.096290995 | 2.920050901 | 3.196104449 | 0.00212489 | 0.024844471 | -1.546839129 |
| GFAP | 1.806210223 | 4.212135544 | 3.196028702 | 0.002125373 | 0.024844471 | -1.547041113 |
| HOPX | 1.104756903 | 3.9946356 | 3.191282318 | 0.002155868 | 0.025049468 | -1.559690787 |
| MT1G | 1.074926414 | 3.738009233 | 3.185883744 | 0.002191051 | 0.025205705 | -1.574062106 |
| PCSK1N | 1.610632018 | 5.216199002 | 3.180837257 | 0.002224426 | 0.025435769 | -1.587480233 |
| L1CAM | -1.062751664 | 8.996618006 | -3.180422123 | 0.002227192 | 0.025436812 | -1.588583351 |
| EPB41L4B | -1.228139133 | 8.18163986 | -3.175529302 | 0.002260043 | 0.025642592 | -1.601576957 |
| ATP6V1G2 | 1.17538672 | 3.969756127 | 3.151256118 | 0.002429833 | 0.026926399 | -1.665823101 |
| ESM1 | -1.135930396 | 4.430029112 | -3.126453434 | 0.002615601 | 0.028212291 | -1.731099334 |
| DUSP26 | 1.014096271 | 3.208586843 | 3.114523329 | 0.002709609 | 0.028746423 | -1.76236273 |
| MGC87042 | -1.150903242 | 6.379822943 | -3.094001214 | 0.002878741 | 0.029897403 | -1.815936338 |
| TMEM155 | -1.375468363 | 7.683574348 | -3.082602006 | 0.002976894 | 0.030516773 | -1.845581535 |
| DPP6 | 1.041261674 | 3.222861081 | 3.076379878 | 0.003031778 | 0.030830097 | -1.861728918 |
| FOXD3 | -1.272013704 | 8.269855903 | -3.065209724 | 0.003132681 | 0.031535763 | -1.890656595 |
| PPP1R1B | 1.365806334 | 3.186667139 | 3.060361482 | 0.003177443 | 0.03183635 | -1.903187951 |
| CRTAM | 1.064753606 | 3.05557917 | 3.0579065 | 0.003200336 | 0.031929477 | -1.909527783 |
| GRIA2 | 1.543995325 | 3.654093443 | 3.050477733 | 0.003270548 | 0.032358349 | -1.928689064 |
| COL3A1 | -1.339862065 | 8.822612199 | -3.049040889 | 0.003284293 | 0.032443698 | -1.932391169 |
| TMEM178 | 1.011708791 | 5.583304606 | 3.035152919 | 0.003419945 | 0.033265401 | -1.96810729 |
| C12orf69 | -1.255916594 | 7.317865036 | -3.021702038 | 0.003556283 | 0.034034366 | -2.002583334 |
| C2orf40 | 1.473185645 | 5.961772961 | 3.006244469 | 0.003719197 | 0.035133753 | -2.0420612 |
| AKR1C1 | 1.010172251 | 4.446891892 | 2.996920928 | 0.003820787 | 0.035662932 | -2.06579962 |
| ADCY1 | 1.108802191 | 4.141734407 | 2.97243043 | 0.004100045 | 0.037178253 | -2.127889789 |
| COL15A1 | -1.192850077 | 8.495625786 | -2.970578613 | 0.004121913 | 0.037269981 | -2.132569027 |
| NEUROD1 | 1.366400354 | 2.546130235 | 2.943939093 | 0.004448707 | 0.039128865 | -2.19963838 |
| LOC402778 | 1.02822239 | 4.732750136 | 2.939272101 | 0.004508372 | 0.039344283 | -2.211341113 |
| GABRD | 1.216006238 | 3.126169722 | 2.933580307 | 0.004582141 | 0.039732782 | -2.225594493 |
| SCN4B | 1.107555579 | 4.544289682 | 2.932152366 | 0.004600822 | 0.039804022 | -2.229167039 |
| TSPAN8 | -2.169280371 | 5.819709622 | -2.920709873 | 0.00475309 | 0.040621895 | -2.257747028 |
| PSD2 | 1.0216975 | 3.047473647 | 2.913281215 | 0.004854426 | 0.041072162 | -2.276256005 |
| C2orf88 | -1.070224946 | 8.723104577 | -2.910414422 | 0.004894065 | 0.041325727 | -2.283389174 |
| FAT2 | 1.056282667 | 2.474458814 | 2.866764998 | 0.005535757 | 0.044577377 | -2.391332488 |
| GPNMB | -1.19361585 | 7.46524446 | -2.840233142 | 0.005962855 | 0.04666241 | -2.456330474 |
| KANK4 | -1.275028669 | 8.517111724 | -2.819431807 | 0.006318715 | 0.048294488 | -2.506962351 |
| C4orf49 | 1.01047225 | 4.726531842 | 2.811829087 | 0.006453584 | 0.048931956 | -2.525395734 |
